# Chordae Rupture Alters Tricuspid Valve Leaflet Biomechanics

**DOI:** 10.1007/s13239-025-00815-9

**Published:** 2026-01-05

**Authors:** Julia Clarin, Keyvan A. Khoiy, Samuel D. Salinas, Dipankar Biswas, Kourosh T. Asgarian, Francis Loth, Rouzbeh Amini

**Affiliations:** 1 Department of Bioengineering, Northeastern University, Boston, MA USA; 2 Department of Biomedical Engineering, The University of Akron, Akron, OH USA; 3 Department of Neurosurgery, Johns Hopkins University School of Medicine, Baltimore, MD USA; 4 Jersey Shore University Medical Center, Hackensack Meridian Health, Neptune City, NJ USA; 5 Department of Mechanical & Industrial Engineering, Northeastern University, Boston, MA USA

**Keywords:** Chordae tendineae rupture, Tricuspid valve, Mechanical strains, Cardiac trauma, Ex vivo porcine heart model, Practice problem

## Abstract

****Purpose**:**

Tricuspid valve chordae tendineae rupture is a valvular lesion that is often overlooked, though is postulated to be more prevalent than currently known. We examined the hemodynamics and biomechanical response of the tricuspid valve leaflets following chordae rupture to understand how acute changes in the post-rupture mechanical environment may contribute to long-term remodeling responses.

****Methods**:**

Porcine valve leaflet deformation was studied in an intact heart in an ex vivo setup using sonomicrometry techniques before and after chordae rupture, which was induced by severing a chordae bundle connected to the septal leaflet.

****Results**:**

Following chordae rupture, pulmonary artery pressure dropped approximately 5 mmHg ($p=0.048$), indicating that valvular regurgitation occurred immediately after rupture. Mean maximum principal stretch of the septal leaflet increased 12% after rupture ($p=0.006$).

****Conclusion**:**

The immediate changes in post-rupture septal leaflet stretches show that chordae tendineae rupture acutely alters the biomechanical environment of the tricuspid valve, which may result in chronic tissue remodeling responses.

****Non-technical Summary**:**

The tricuspid valve is one of the four valves in the heart. Rupture of supporting structures of the tricuspid valve leaflet, known as chordae tendineae, may be more common than previously thought. In this study, we used excised pig hearts to examine how chordae rupture affects valvular function. With our experimental beating heart system, we pumped fluid through the hearts under realistic conditions and measured changes in pressure and leaflet motion before and after chordae rupture. After rupture, we observed a change in pressures and leaflet motion, causing the valve to leak and become less efficient. These changes may influence how the valve functions over time.

**Supplementary Information:**

The online version of this article (10.1007/s13239-025-00815-9) contains supplementary material, which is available to authorized users.

## Introduction

Tricuspid valve (TV) regurgitation arises from insufficient leaflet coaptation that may result from a number of organic lesions and/or secondary diseases [1]. TV regurgitation poses a great long-term risk to the patient since it may be well tolerated in early stages and the patient may appear asymptomatic [2–7]. However, as the condition progresses undetected, the likelihood of right ventricular dysfunction and atrial fibrillation rises, ultimately contributing to chronic heart failure and increased morbidity and mortality [2, 8].

The chordae tendineae play a critical role in TV function, serving as fibrous connections between the leaflets and ventricular papillary muscles. The chordae tendineae facilitate proper opening and closure of the valve. When ruptured, the chordae tendineae can no longer prevent leaflet flail into the right atrium, leading to valvular insufficiency [9]. Chordae tendineae rupture is most commonly associated with severe blunt chest trauma (e.g., car accidents) [4, 5, 10–17], but can also occur as a rare complication of medical procedures like right heart catheterization [18, 19] or from degenerative connective tissue disorders such as Marfan syndrome [20].

Between 1999 and 2008, over 28,000 operations involving the TV were performed in the US [21]. In the case of traumatic TV regurgitation, chordae rupture was cited as the most common underlying cause [22, 23], with one study placing the incidence at 55% of patients [2]. However, TV regurgitation is frequently underdiagnosed or only identified during evaluation for unrelated conditions [3, 24]. Previous clinical reports have emphasized the subtle clinical manifestations and the underdiagnosis of this critical condition [25, 26]. While TV regurgitation can be properly diagnosed via transthoracic echocardiography and transesophageal echocardiography, it is usually concurrent with the diagnosis of another condition [3, 24]. Consequently, long delays between the onset of chordae rupture and its detection–over a decade in several cases [27, 28]—are alarmingly common [2, 3, 5, 6]. The risks associated with untreated TV regurgitation [8, 29] coupled with the importance of early treatment and intervention [30] underscore a critical need for a deeper understanding of chordae rupture and its impact on valve function. Additionally, it is not well understood why asymptomatic and tolerable regurgitation would become exacerbated over time.

We have previously characterized the mechanical and microstructural responses of the TV to mechanical loading [31–35], and have shown that the microstructural architecture of the TV leaflet extracellular matrix is highly dependent on the biomechanical environment to which the leaflets are subjected [36]. We have also demonstrated that the mechanical microenvironment of TV interstitial cells is highly influenced by the structure and composition of the extracellular matrix in addition to macro-scale biomechanical loading [37–39]. Other investigators have shown that acute biomechanical changes in cardiac valves can lead to remodeling responses that could negatively affect the mechanical integrity of the valve extracellular matrix [40–42]. As such, one could postulate that after chordae rupture, the biomechanics of the TV leaflets are altered, triggering mechanobiological responses in the valve extracellular matrix that can manifest at the tissue level. These responses may eventually exacerbate valvular dysfunction and the initial asymptomatic or tolerable regurgitation over time. The complex relationship between the tissue’s mechanical environment, microstructural remodeling behavior, and subsequent valvular function offers a potential mechanistic explanation for the delay between the onset of chordae rupture and the worsening regurgitation observed clinically.

In our previous study, we found that chordae ruptured affected the dynamic deformation of the tricuspid annulus in ex vivo porcine hearts [43]. In the present study, we aimed to characterize mechanical changes in the TV septal leaflet following chordae tendineae rupture to better understand the acute impacts on TV biomechanics.

## Methods

### Ex Vivo Beating Heart System

**Fig. 1:**
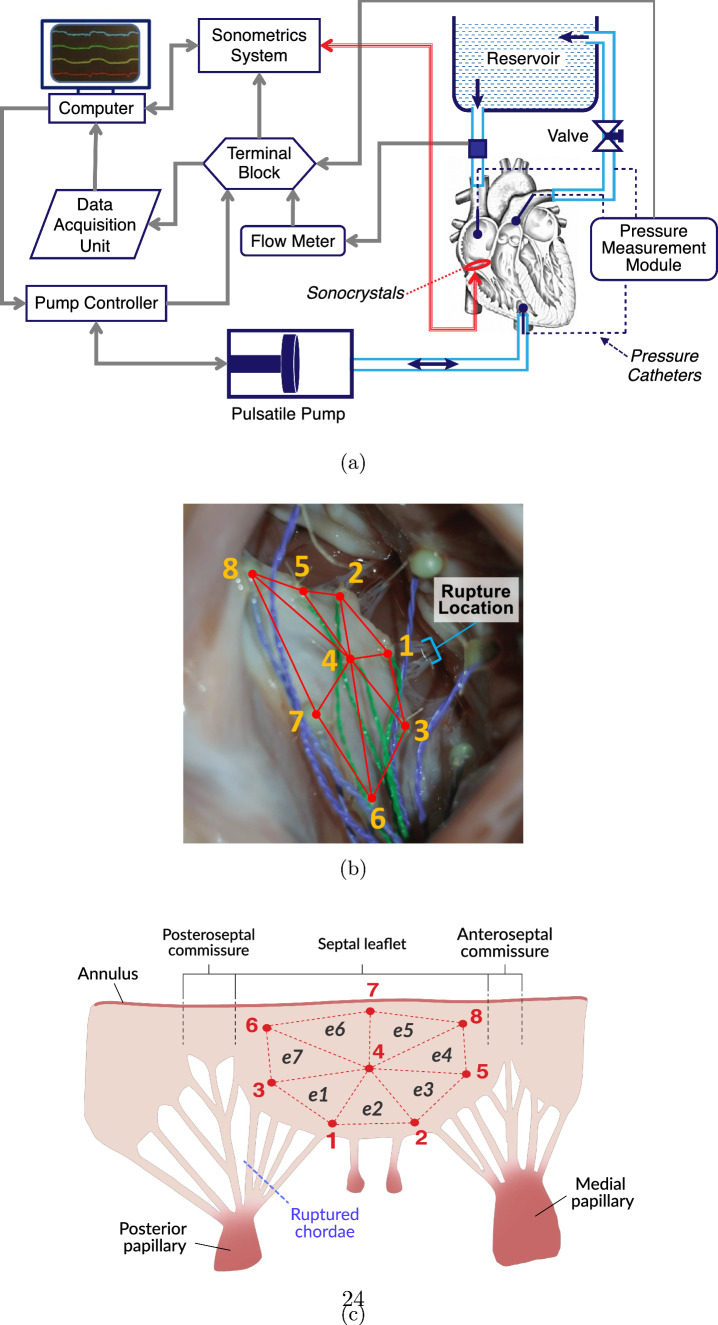
**a** Schematic of the ex vivo beating heart system. **b** Arrangement of sonocrystal nodes on the septal tricuspid leaflet (STL) surface viewed through the superior vena cava. To induce rupture, a chordae bundle was severed at the leaflet’s free edge, indicated by the blue bracket near node 1. **c** Schematic of sonocrystal nodes with respect to nodes 6 through 8 were placed along the annular edge of the STL. Triangular elements used for analysis are outlined in red, with vertices indicating sonocrystal node placement

In a previously developed ex vivo passive beating heart system (Fig. 1a), a pulsatile hydraulic piston pump (SuperPump AR Series, Vivitro Labs, Inc., Victoria, BC, Canada) was utilized to beat porcine hearts passively by circulating phosphate-buffered saline (PBS) into the hearts [43, 44]. The resulting transvalvular pressure from this circulation forced the tricuspid valve to open and close, imitating the native deformations of intact TV leaflets. The apparatus was developed in a way that allowed the monitoring and recording of right atrial pressure (RAP), right ventricular pressure (RVP), and pulmonary artery pressure (PAP) throughout the cardiac cycle. The pump was set to a standard 70 bpm waveform in compliance with the guidelines for heart valve testing by the International Standard Organization (ISO 5840) [45]. Other pump parameters were set to keep the hydrodynamic pressures in the TVs in the test setup consistent with those in a native valve during the cardiac cycle. More details about this experimental setup can be found in our previous publication [44].

### Sample Preparation

Fresh porcine hearts were obtained from a local abattoir (3-D Meats, Dalton, OH) and maintained in chilled PBS during transport. Hearts were tested within 2–6 h post-mortem to avoid any changes in the time-dependent biomechanical response of the leaflets [46]. The right-sided heart chambers were flushed with PBS and carefully examined with an endoscopic camera (an SSVR-710 Snakescope) to ensure the chambers and valves were free of blood clots. Similar to previous biomechanical studies of cardiac valves, sonomicrometry techniques (Sonometrics Co., London, ON, Canada) were used to quantify leaflet motion over the cardiac cycles [47, 48]. Eight sonocrystals (Sonometrics Co., London, ON, Canada), 1 or 2 mm in diameter, were sutured over the septal leaflet in a predefined arrangement (Fig. 1b). Sonocrystal nodes 1, 2, 3, and 5 were placed along the free edge of the septal leaflet, node 4 was located at the center of the leaflet, and nodes 6, 7, and 8 were placed on the septal section of the tricuspid annulus (Fig. 1c). Specifically, sonocrystal nodes 6 and 8 were placed near the posteroseptal commissure and anteroseptal commissure, respectively (Fig. 1c).

To eliminate the risk of puncturing the heart chambers, suturing was performed through the superior vena cava and the sonocrystal wires were passed through the inferior vena cava. The septal leaflet was selected due to its position immediately below the superior vena cava that provided ease of access during suturing. After securing all sonocrystals, the inferior vena cava and coronary vein were clamped shut to prevent leakage during experiments. The heart was then connected to the ex vivo beating heart apparatus through the superior vena cava, pulmonary artery, and an incision made at the right ventricular apex as described previously [44]. Three 3-mm-diameter sonocrystals were externally attached around the apex to create a consistent reference frame for analyzing positional data.

### Data Acquisition

A sonomicrometer (TRX Series 16, Sonometrics Co., London, ON, Canada) was utilized to trigger the sonocrystals and record their signals. The sonomicrometer communicated with a computer through a USB port using SonoLabDS3 software (Sonometrics Co., London, ON, Canada). Catheter-type pressure probes (SPR-524 and PCU-2000, AD Instruments, Colorado Springs, CO) were inserted through access points in the right atrium, right ventricle, and the pulmonary artery to measure RAP, RVP, and PAP, respectively (shown by dotted lines in Fig. 1a). To simplify data synchronization, the pressure sensors were connected to the analog input channels of the sonomicrometer. Once the pump was running, an endoscopic camera (SSVR-710 Snakescope) was sent into the right atrium through a probe embedded in the superior vena cava connector to visually assess the TV leaflet coaptation with each simulated heartbeat [44]. An endoscopic recording of TV leaflet motion under simulated beating heart conditions for the intact condition is included in Supplementary Materials. Prior to recording data, an oscilloscope (MSO2014B, Tektronix, Beaverton, OR) was used to visualize pressure ranges and fine-tune sonocrystal sensitivity to reduce the signal-to-noise ratio. After adjusting the necessary parameters, pressures and sonocrystal signals were recorded in SonoLabDS3 software at a frequency of 100 Hz (according to ISO 5840 requirements) for 20 s, equivalent to approximately 23 cardiac cycles. Flow rate was measured using a transonic flowmeter (T108, Transonic Systems, Inc., Ithaca, NY) at the inflow tube proximal to the heart (Fig. 1c).

After recording data for the intact condition, the pump was briefly stopped to access the chordae tendineae of the septal leaflet. To model rupture, the septal leaflet chordae bundle adjacent to the posteroseptal commissure (see Fig. 1c) was severed with surgical scissors. Right heart pressurization was restored immediately after completing the simulated rupture, and leaflet coaptation was visually assessed with the endoscopic camera. Data were then recorded for the ruptured condition using the same parameters and settings. Sonocrystal sensitivity remained consistent between intact and ruptured conditions and only required adjustments between different samples. In total, eight successful experiments were conducted.

### Pressure Data Processing and Analysis

Hemodynamic data (RAP, RVP, PAP) were recorded simultaneously with deformation data in SonoLabDS3 (Sonometrics Co., London, ON, Canada), ensuring automatic synchronization for both intact and ruptured conditions. To eliminate the effects of hydrostatic pressure due to elevation differences between the pressure probes and the fluid surface (see Fig. 1a), average pressure signals were adjusted. Specifically, RAP and RVP were shifted by subtracting the hydrostatic pressure equivalent, such that their diastolic values approached zero, with a minor adjustment to the corresponding PAP. This effectively removed the pressure offset caused by vertical height differences: from the reservoir fluid surface to the right atrium (for RAP and RVP) and from the right ventricle to the pulmonary artery (for PAP). These corrections helped to ensure consistent and accurate pressure curves given the relatively low pressure levels on the right side of the heart.

To ensure consistency across samples, the start of the cardiac cycle was aligned with the standard 70 bpm waveform of the pump, with systole approximated to occur between 0.3 and 0.5 s. Processed RAP, RVP, and PAP signals were averaged across samples at each time point to generate representative curves of intact and ruptured conditions. For statistical comparison, mean systolic and diastolic pressures were computed for each sample before and after rupture. The timing of tricuspid valve opening and closure was estimated from the intersection points of the average RVP and RAP curves. Similarly, pulmonary valve (PV) opening and closure times were identified from the intersections of the average RVP and PAP curves. These features were extracted descriptively and were not used for statistical testing.

### Deformation Data Processing and Analysis

The sonocrystal signals were recorded throughout the experiment, providing the position of each sonocrystal relative to one another. Because each sonocrystal functions as both a transmitter and receiver, the raw signal data were processed in SonoVIEW software (Sonometrics Co.) to filter noise and identify any signal drift. With SonoXYZ software (Sonometrics Co.), the processed signals were used to reconstruct the absolute positional coordinates of each sonocrystal with respect to a defined coordinate system throughout the entire cardiac cycle. These positional data, which represent the deformation of the septal leaflet surface during the cardiac cycle, were utilized to calculate the strains over the septal leaflet surface for both intact and ruptured conditions [44, 49–51].

Triangular planar elements $e1{-}e7$ were defined between sonocrystal nodes as shown in Fig. 1c. Eulerian strains were calculated for each element at every time frame with respect to the intact reference frame at minimum RAP for both conditions. For directional strains, the global radial direction vector was defined from sonocrystal node 4 (center of leaflet) to node 7 (center of septal leaflet annulus), and the global circumferential direction vector was defined from nodes 6 to 8 along the annular edge of the leaflet (Fig. 1c). More information regarding the triangulation and strain calculation can be found in our previous publication [44]. Strain results for corresponding triangular regions were averaged across samples and plotted on a representative leaflet geometry for visual purposes.

### Statistical Analysis

The sonocrystal positions and pressure signals were recorded for 20 s, equivalent to approximately 23 cardiac cycles. Variability in positional data and pressure signals was minimal across cardiac cycles, evidenced by a standard deviation-to-mean ratio of less than 1% when peak values of individual cycles were compared. To further reduce any signal noise, the synchronized displacement and pressure signals were averaged over all cardiac cycles, and these average signals were used for subsequent calculations and analysis.

For statistical comparison of hemodynamic data, mean systolic and diastolic pressures were computed for intact and ruptured conditions. Briefly, systole was defined from approximately 0.3 to 0.5 s, with diastole spanning the remainder of the cardiac cycle. Pairwise comparisons of intact and ruptured pressures (RAP, RVP, and PAP) during the systolic and diastolic phases were conducted via paired t-tests with a significance threshold of $\alpha = 0.05$. For statistically significant results, a Bonferroni correction was applied to account for multiple comparisons (two phases per pressure).

For comparison of intact and ruptured mechanical data, a two-way repeated-measures analysis of variance (ANOVA) was performed with within-subject factors of condition (intact vs. ruptured) and region (seven leaflet locations), treating each heart (n = 8) as a repeated measure. Sphericity was assessed using Mauchly’s test, and Greenhouse–Geisser corrections were applied when the assumption of sphericity was violated. Statistical significance was defined as $p < 0.05$. When significant main or interaction effects were identified, post hoc pairwise comparisons were conducted using Bonferroni-adjusted tests to account for multiple comparisons.

Results are reported as mean ± standard error of the mean (SEM). Statistical analyses were performed using *jamovi* (Version 2.6; The jamovi project, 2024 [52]) and *R* (Version 4.4; R Core Team, 2024 [53]) with the *afex* package [54].

## Results

### Hemodynamics

**Fig. 2:**
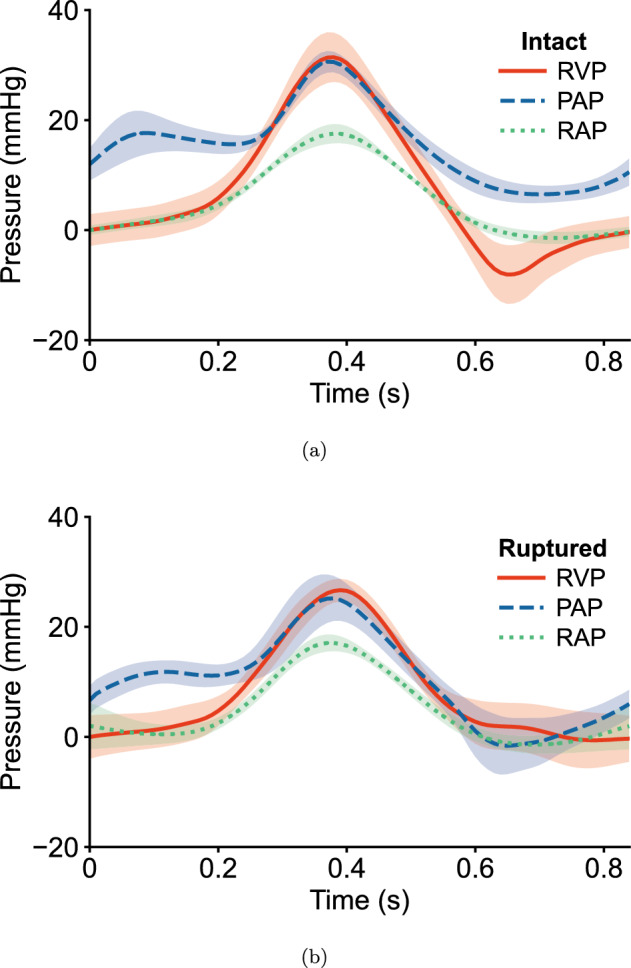
Average hemodynamic pressures during the cardiac cycle for **a** intact and **b** ruptured cases. Curves show right ventricular pressure (RVP), pulmonary artery pressure (PAP), and right atrial pressure (RAP) with shaded regions indicating SEM. Maximum RVP dropped by 5 mmHg post-rupture. Mean systolic and diastolic PAP were significantly altered post-rupture (p < 0.05)

Figure 2 illustrates the average pressure signals measured before (Fig. 2a) and immediately after chordae rupture (Fig. 2b). For the intact condition (Fig. 2a), diastolic RVP and RAP remained close to zero and reached peak systolic pressures of 32 mmHg and 18 mmHg, respectively. Peak RVP occurred at approximately 0.37 s. PAP ranged from approximately 5 to 32 mmHg. With the exception of a slight negative RVP early in the diastolic phase due to hydrostatic pressure corrections, the RVP range closely matched those reported in porcine hearts [55–58]. The observed peak systolic RAP of 18 mmHg exceeds typical ranges for the human heart; however, the overall RAP and PAP signals closely align with those reported in healthy human hearts [59]. Few data exist on healthy porcine right heart pressures [60], limiting comparisons to healthy porcine pressures. The corresponding standard errors (shown by shaded areas in Fig. 2) were small, indicating relatively consistent pressure curves despite variability between individual porcine hearts.

Post-rupture pressure signals are presented in Fig. 2b. After chordae rupture, minimum and maximum RVP values ranged from approximately 0 to 27 mmHg, RAP from 0 to 18 mmHg, and PAP from nearly 0 to 26 mmHg. Mean systolic and diastolic pressures before and after rupture are shown in Table 1. Paired comparisons of mean systolic and diastolic pressure signals revealed a statistically significant difference between intact and ruptured PAP during both systolic ($p = 0.024$) and diastolic ($p = 0.042$) phases. Changes in RVP and RAP were not found to be significant for either phase. After applying Bonferroni correction to account for multiple comparisons, the reduction in diastolic pressure following rupture was no longer statistically significant ($p = 0.084$), while the systolic difference remained significant ($p = 0.048$).

Closer inspection of the average RVP and RAP pressure signals in Fig. 2a indicates that the TV closed around 0.2 s and opened around 0.55 s. Whereas for the ruptured condition (Fig. 2b), the same intersections of the RVP and RAP signals were not clearly discernible; thus, the time points of TV closure and opening could not be specified. For the PV, the intersections of the average RVP and PAP signals in Fig. 2a indicate that the PV opened and closed at approximately 0.3 s and 0.45 s, respectively. After chordae rupture (Fig. 2b), the PV was estimated to open at approximately 0.3 s and close around 0.5 s. While statistical comparisons were not conducted on the timing of valve events, the loss of distinct curve intersections associated with TV function post-rupture qualitatively reflects altered pressure dynamics.

### Leaflet Stretch During the Cardiac Cycle

**Fig. 3:**
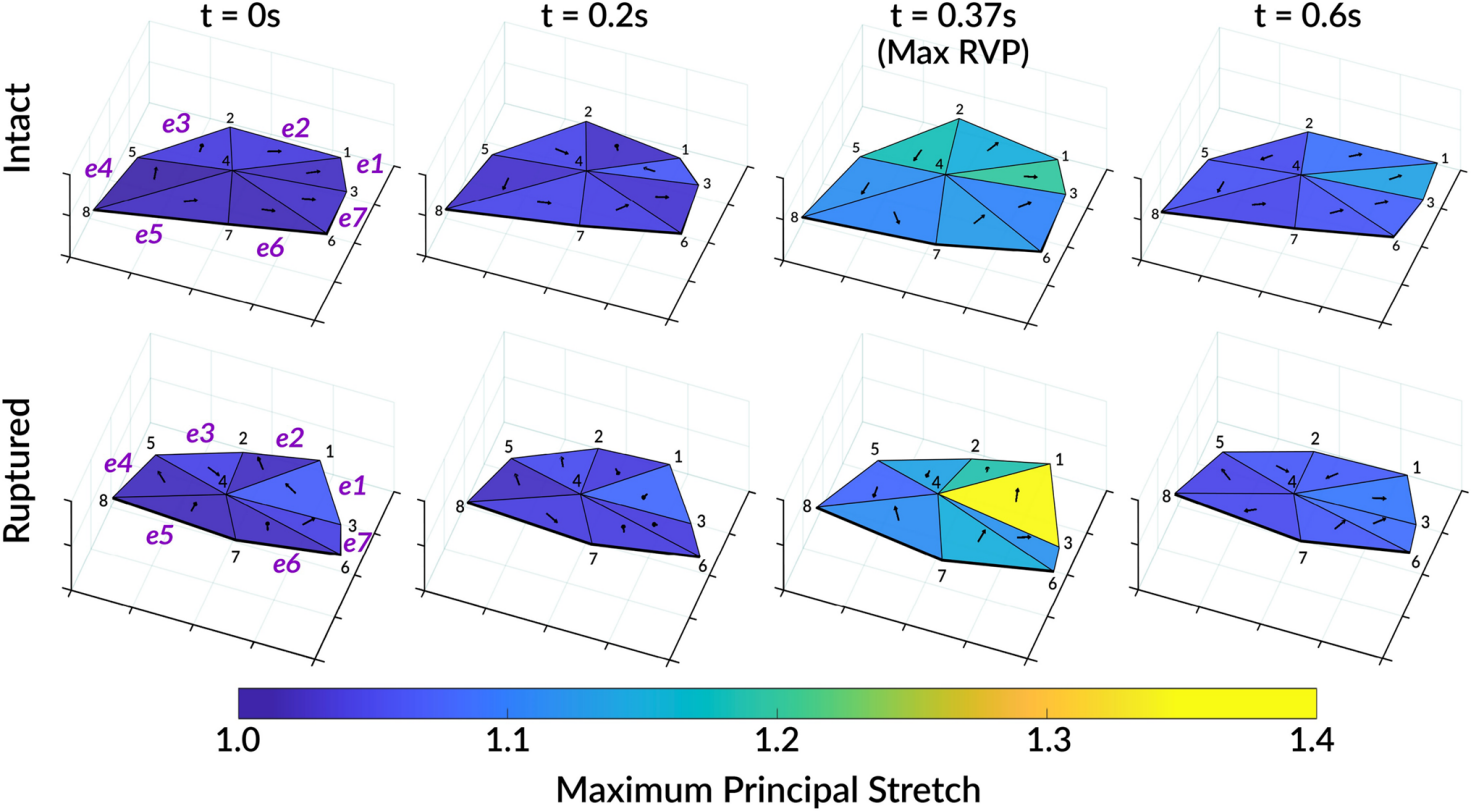
Mean maximum principal stretches across the septal leaflet surface before and after chordae rupture at multiple time points in the cardiac cycle, shown on a representative leaflet geometry. Arrows depict the principal direction for the selected geometry, sonocrystal nodes are labeled in black text, and elements $e1{-}e7$ are labeled in bold purple text for t = 0 s. Overall maximum principal stretch significantly increased by $0.12 \pm 0.03$ ($p=0.006$) at maximum right ventricular pressure (Max RVP)

The dynamic deformation of the septal leaflet is depicted in Fig. 3, where mean maximum principal stretches are shown on a representative leaflet geometry at four key time points in the cardiac cycle–mid- to late diastole (0.0 s), late diastole (0.2 s), peak systole (0.37 s), and early diastole (0.6 s)–both before and after chordae rupture. Peak maximum principal stretch occurred near the time of maximum RVP ($t \approx 0.37$ s). As shown for the representative geometry in Fig. 3, the ruptured leaflet configuration was visibly altered along the free edge near the point of rupture and most pronounced at peak RVP, consistent across all samples.

To test for statistical significance, a two-way repeated-measures ANOVA was conducted with within-subject factors of *condition* (intact vs. ruptured) and region (seven leaflet locations) for the maximum principal stretch at each time point shown in Fig. 3. Sphericity was evaluated using Mauchly’s test, and Greenhouse–Geisser corrections were applied where assumptions were violated.

At baseline ($t=0$ s), no significant differences in maximum principal stretch were observed between intact and ruptured conditions, and neither regional nor interaction effects reached significance. By early systole ($t=0.2$ s), a significant main effect of condition emerged [$F(1,7)=11.86$, $p=0.011$, $\eta ^{2}_{p}=0.63$], indicating higher stretches following chordae rupture, whereas regional ($p=0.108^{\dagger }$) and condition–region ($p=0.114^{\dagger }$) effects were not significant.^1^

At maximum RVP, the ANOVA showed significant main effects of rupture [$F(1,7)=15.05$, $p=0.006$, $\eta ^{2}_{p}=0.68$] and region ($p=0.028^{\dagger }$) at maximum RVP, as well as a significant condition–region interaction ($p=0.022^{\dagger }$), indicating a global increase in maximum principal stretch after rupture with region-dependent magnitude. Post hoc Bonferroni-corrected comparisons confirmed a significant overall increase in maximum principal stretch of $0.12 \pm 0.03$ ($p=0.006$). Although no individual regional differences remained significant after correction, mean values were highest near the free edge (region *e*1), consistent with local leaflet elongation and loss of chordal support.

This difference persisted at $t=0.6$ s, corresponding to the diastolic phase, with a significant main effect of condition [$F(1,7)=6.93$, $p=0.034$, $\eta ^{2}_{p}=0.50$] and region ($p=0.044^{\dagger }$), suggesting increased stretch magnitude across the leaflet with spatial variability but without a significant interaction.

### Directional Strains at Peak Systole

**Fig. 4:**
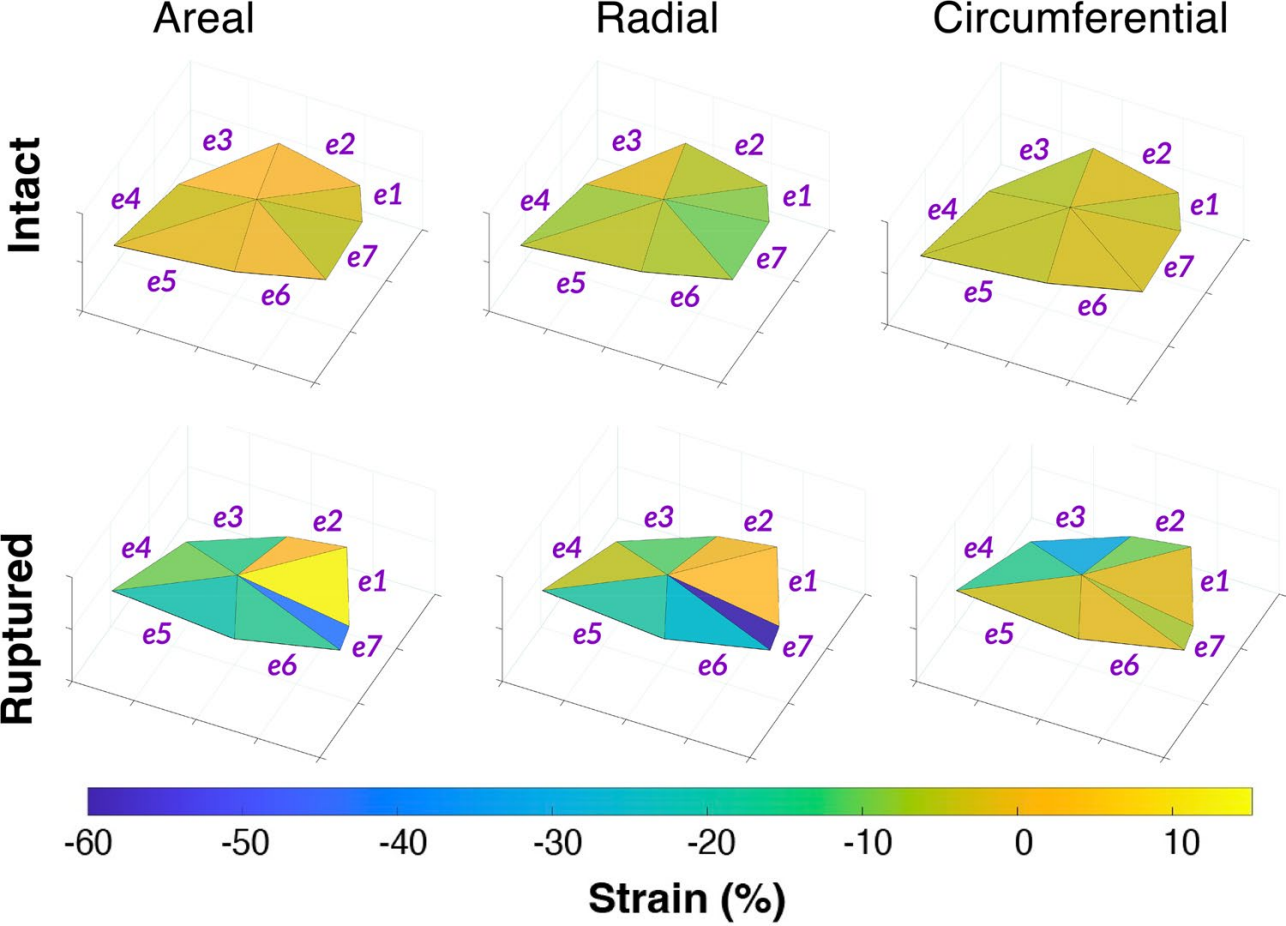
Spatial distribution of areal, radial, and circumferential strains at maximum right ventricular pressure (RVP) before and after chordae rupture. Strains represent the means for $n=8$ samples displayed on a representative leaflet geometry. Elements $e1{-}e7$ are labeled in bold purple text. Ruptured areal, radial, and circumferential leaflet strains significantly decreased at maximum RVP

Figure 4 compares the spatial distributions of areal, radial, and circumferential strains at maximum RVP ($t = 0.37$ s) before and after chordae rupture, plotted on the same representative geometry shown in Fig. 3. The strains represent the average across all samples. The radial direction was defined between the annulus and leaflet free edge (between nodes 4 and 7), while the circumferential direction aligned with the annulus (from nodes 6 to 8). Positive strain magnitudes indicate extension, and negative magnitudes indicate contraction along the corresponding direction.

Two-way repeated-measures ANOVA revealed distinct effects of chordae rupture on leaflet deformation at peak RVP across all three directional strain components—radial, circumferential, and areal—with *condition* (intact vs. ruptured) and *region* (seven leaflet locations) as within-subject factors. Sphericity was evaluated using Mauchly’s test, and Greenhouse–Geisser corrections were applied where necessary.

**Areal strain** at maximum RVP decreased significantly following rupture [$F(1,7)=17.65$, $p=0.004$, $\eta ^{2}_{p}=0.72$], with post hoc Bonferroni-corrected comparisons confirming a significant overall reduction in areal strain of $14.0\% \pm 3.0\%$ ($p=0.004$), consistent with decreased areal expansion after rupture. Mean spatial strain patterns suggested lower areal strains near the rupture location (*e*1) and along the annular edge of the leaflet ($e4{-}e6$), though the main effect of region ($p=0.057^{\dagger }$) and the condition–region interaction ($p=0.129^{\dagger }$) were not significant after correction.^2^

**Radial strain** averaged $1.9\% \pm 1.3\%$ at maximum RVP for the intact leaflet and decreased to $-10.2\% \pm 5.9\%$ following rupture. The ANOVA revealed a significant main effect of rupture [$F(1,7)=10.80$, $p=0.013$, $\eta ^{2}_{p}=0.61$], whereas regional ($p=0.077^{\dagger }$) and interaction ($p=0.146^{\dagger }$) effects were not significant after correction, suggesting a predominantly uniform radial response across the leaflet. Post hoc Bonferroni-adjusted tests verified an overall decrease of $12.0\% \pm 4.0\%$ in radial strain ($p=0.013$), indicating reduced radial extension after rupture.

**Circumferential strain** at maximum RVP decreased from $4.80\% \pm 0.80\%$ to $-2.20\% \pm 2.5\%$ post-rupture. The ANOVA confirmed a significant main effect of rupture [$F(1,7)=14.12$, $p=0.007$, $\eta ^{2}_{p}=0.670$], along with significant region ($p=0.022^{\dagger }$) and interaction ($p=0.014^{\dagger }$) effects, demonstrating spatial variation in strain as well as region-dependent responses to rupture. Mean strain maps (Fig. 4) showed the greatest decreases near the rupture site (*e*1) and the adjacent free-edge region ($e2{-}e3$). Post hoc Bonferroni-corrected comparisons confirmed an overall decrease in circumferential strain of $7.00\% \pm 2.0\%$ ($p=0.007$). Although individual regional differences were not significant after correction, mean trends indicated greater decreases near the rupture location (*e*1) and the leaflet free edge ($e2{-}e3$), suggesting localized reductions in circumferential contraction.

### Leaflet Mechanics Across the Cardiac Cycle

**Fig. 5:**
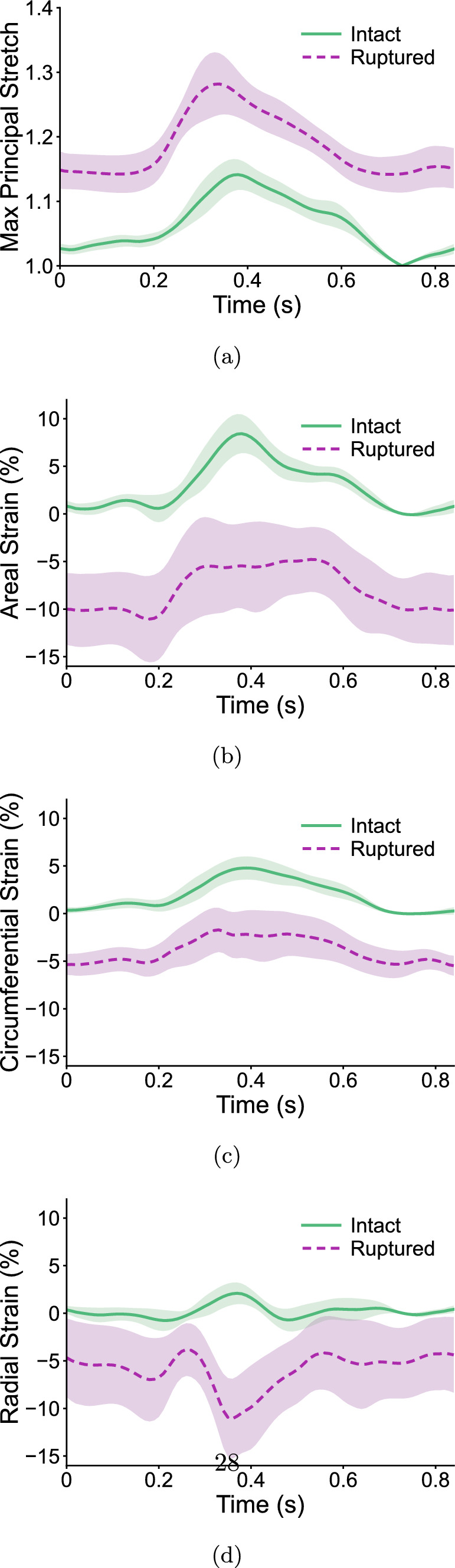
Distributions of mean **a** maximum principal stretch, **b** areal strain, **c** circumferential strain, and **d** radial strain across the septal leaflet surface (n = 8) throughout the cardiac cycle before and after rupture. Shaded regions indicate standard error

To characterize septal leaflet stretch and strain throughout the cardiac cycle, global mean values ± SEM ($n=8$) were computed by averaging strain across all seven leaflet elements at each time point. Figure 5 shows the resulting mean curves for the maximum principal stretch and areal, circumferential, and radial strains for intact and ruptured conditions throughout the cardiac cycle. As shown by the shaded regions in Fig. 5, standard errors were consistently larger following rupture, indicating greater inter-sample variability. The post-rupture maximum principal stretch curve maintained a similar shape to the intact case, but the magnitudes were notably elevated (Fig. 5a).

Conversely, an overall negative shift was observed for ruptured areal, circumferential, and radial strain curves. Comparison of these plots with the pressure plots in Fig. 2 shows that peak maximum principal stretch, areal strain, and circumferential strain occurred near the time of maximum RVP (approximately 0.4 s). Interestingly, the post-rupture radial strains rapidly decreased around 0.4 s, which was in contrast to the slight increase observed in intact radial strains (Fig. 5d).

## Discussion

Our ex vivo beating heart system was able to reproduce ventricular pressure waveforms that closely match those of an active heart [59]. While caution should be taken when interpreting animal data for human pathophysiology, the pulmonary hemodynamics of human and porcine hearts are relatively similar [55–59, 61, 62]. Additionally, the structural and biomechanical characteristics of porcine valve tissue are comparable to those of human valves [44, 63–66]. Hence, studying porcine TV deformations with our ex vivo system can provide more relevant information regarding the deformations of the TV in a human heart as compared to rodent models [67]. Moreover, our experimental model provides advantages over in vivo animal models and human clinical studies, as it enabled direct measurement of the acute changes in flows, pressures, and leaflet deformation pre- and post-rupture within the same heart.

In this study, septal leaflet deformations and surface strains were calculated throughout the cardiac cycle using sonomicrometry techniques (Fig. 1) [44]. Right heart pressures, leaflet deformations, and strains were measured before and after chordae rupture, which was induced by severing the septal chordae adjacent to the posteroseptal commissure (Fig. 1c). Immediately following chordae rupture, a significant decrease in pulmonary artery pressure occurred ($p = 0.048$; Fig. 2; Table 1), suggesting the presence of regurgitation post-rupture. This outcome is consistent with previous reports showing that chordal disruption impairs valve competence and reduces effective forward ejection into the pulmonary circulation [2–7, 18].

Across all mechanical metrics, the ANOVA results consistently demonstrated that chordae rupture altered overall septal leaflet deformation, with post hoc Bonferroni-corrected comparisons confirming a significant increase in maximum principal stretch (Fig. 3), and significant reductions in areal, circumferential, and radial strain at peak RVP (Fig. 4). Significant regional and interaction effects for maximum principal stretch and circumferential strain indicated spatially heterogeneous responses, with the greatest changes near the free edge and anterior–septal junction. Radial and areal strains exhibited more uniform, global responses across the leaflet surface. Although individual regional comparisons did not remain significant after correction—likely due to the small sample size and conservative adjustment—the pattern of means indicated localized effects, with the greatest rupture-induced changes typically observed in regions near the leaflet free edge and rupture location (*e*1 and *e*2).

As shown in Fig. 5, a trend in elevated maximum principal stretch was observed throughout the cardiac cycle, whereas areal, circumferential, and radial strain magnitudes exhibited a decreasing trend post-rupture. Notably, radial strains exhibited a sharp negative trend at peak right ventricular pressures, opposite to our observations for the intact case (Fig. 5d). This post-rupture behavior also contrasts with radial strain patterns reported for healthy tricuspid valve leaflets in beating ovine hearts [68], further suggesting that the altered strain distributions observed in this study were caused by rupture and the subsequent lack of chordal restraint along the leaflet free edge. While limited data exist for the in vivo strains of the porcine TV leaflets, the values obtained in this study are comparable to those reported for the ovine mitral and tricuspid valves [68, 69].

Together, these acute changes in septal leaflet stretches and strains indicate that chordae rupture produces significant global and spatially nonuniform changes in leaflet deformation, consistent across multiple mechanical metrics. Specifically, chordae rupture led to reduced circumferential and radial extension and diminished areal expansion at peak systolic pressures. The spatially heterogeneous strain patterns suggest a redistribution of mechanical loading across the leaflet following loss of chordal support, with localized changes concentrated near the rupture site and free-edge regions.

These tissue-level mechanical changes are expected to significantly alter the mechanical microenvironment in which the extracellular matrix and valve interstitial cells reside [35, 37, 39]. Such changes may elicit a remodeling response within the leaflet tissue microstructure, further influencing leaflet deformation and valve function. In the context of chordae rupture, the altered mechanical environment of the TV tissue may lead to maladaptive microstructural remodeling responses and contribute to the progression of valvular dysfunction or regurgitation.

### Limitations

Due to the inherent differences between the in vivo heart and our ex vivo setup, interpretation of the presented results must take the following limitations into consideration. Specifically, the absence of active myocardial contraction and interaction between the right and left sides of the heart may lead to differences in ventricular and annular shape, particularly during systole [43, 70–72]. Moreover, studies have shown that, due to the lack of perfusion and metabolic activities, the mechanical properties of ex vivo soft tissues can differ from those in the native in vivo environment [73]. To minimize degradation of the ex vivo tissues and avoid introducing experimental artifacts, samples were prepared and both intact and ruptured data were collected within 5 h [46]. The reasonable agreement with TV strain patterns measured in beating ovine hearts [68, 69] supports the validity of our experimental findings.

Other limiting factors such as the method of pressurization and the use of sonomicrometry techniques must also be considered. For example, the alignment of peak RAP with peak RVP and PAP in the intact condition (Fig. 2) may suggest the presence of mild tricuspid regurgitation in normal human physiology. However, due to the nature of our ex vivo system, the simultaneous peak pressures likely reflect differences in the compliance of the experimental system and the method of pressurization with the Vivitro Labs SuperPump (Victoria, BC, Canada). Further, Fig. 2 represents the average of all eight samples, which may obscure small timing offsets between individual traces. In future studies, incorporating a continuous flow meter would enable estimation of regurgitant volume, offering another physiologically relevant metric to assess valve competence.

Sonocrystals were placed in a predefined arrangement as consistently as possible, though variations in septal leaflet anatomy should be considered when interpreting regional strains. Additionally, the weight of the sonocrystals and the resistance of the attached wires could have affected leaflet coaptation. However, we expect that any interference in valve closure due to the sonocrystals was minimal given that leaflet coaptation was visually confirmed via endoscope prior to data collection. Further, no changes were made to the sonocrystals between intact to ruptured measurements so any interference due to the sonocrystals would be consistent across both conditions. Since the results presented in this study center around the changes after rupture rather than the magnitudes themselves, these experimental factors do not invalidate the conclusions of this study. Moreover, sonomicrometry remains the ”gold standard" for capturing leaflet deformation with sub-millimeter accuracy ($< 12\,\upmu$m), and often serves as a benchmark for validating imaging-based methods [68, 71, 74]. Nonetheless, we acknowledge that imaging approaches may complement our sonomicrometry-derived results in future studies.

Lastly, it must be noted that our acute model does not capture the potential long-term remodeling effects resulting from sustained tricuspid regurgitation [8, 29, 75]. However, by isolating the immediate biomechanical response of the leaflet after chordae rupture, this study provides important insights into the early mechanical triggers that may contribute to chronic remodeling processes.

## Supplementary information

(1) Transvalvular pressure traces for the intact valve and after chordae rupture; (2) endoscopic video of the intact valve, viewed from the atrial side.

## Conclusions

In summary, a novel ex vivo apparatus mimicking physiological right heart flows was used in combination with sonomicrometry techniques to examine the effects of chordae tendineae rupture on the deformation of the septal tricuspid leaflet in whole, passively beating ex vivo porcine hearts. Experiments were conducted in eight porcine hearts before and immediately after severing the septal chordae tendineae bundle adjacent to the posteroseptal commissure (Fig. 1c). The deformation of the septal leaflet was altered post-rupture (Fig. 3), and analysis of regional surface strains revealed an appreciable change in maximum principal stretch across the leaflet surface at peak systolic pressure (Fig. 4). Notably, the maximum principal leaflet stretch at peak right ventricular pressure increased by $0.12 \pm 0.03$ ($p=0.006$). Long-term remodeling responses are expected to occur as a result of such changes in the leaflet’s mechanical environment, which may further alter the mechanical behavior and function of the tricuspid valve. Finally, we emphasize the limitations inherent in using an ex vivo setup. Application of the results presented in this study must consider the ex vivo nature of such results, as well as potential differences between porcine and human hearts. The outcomes of this study offer new mechanical insights into the effects of chordae rupture and further highlight the key relationship between tricuspid valve biomechanics and valve function [7, 63, 76–78, 78–83].

**Table 1:** Right heart pressures before and after chordae rupture

Pressure (mmHg)	Mean systole		Mean diastole	
	Intact	Ruptured	Intact	Ruptured
RAP	14.9 ± 0.68	14.2 ± 0.74	3.13 ± 0.57	2.65 ± 0.52
RVP	25.3 ± 1.5	22.6 ± 1.1	3.41 ± 1.1	4.57 ± 0.73
PAP	25.5 ± 1.2	20.9 ± 1.0*	13.3 ± 0.64	7.83 ± 0.77

Mean pressures ± SEM in the right atrium (RAP), right ventricule (RVP), and pulmonary artery (PAP). Asterisks denote significant differences post-rupture (*$p<0.05$)

## Supplementary Information

Below is the link to the electronic supplementary material.

(MP4 41162 kb)

(PDF 17 kb)

## Data Availability

All data supporting the findings of this study are available within the manuscript. Additional materials, including raw data and analysis code, are available from the corresponding author upon reasonable request. Not applicable.

## Appendix: Educational Component

In our recent work [35, 39, 46, 84–90], we have integrated educational components to broaden the impact and accessibility of our research. Along the same lines, the “practice exercise" presented below aims to help students assess their grasp of the biomechanics of soft tissues while using some of the data presented in this manuscript.

### Practice Exercise

Heart valves, like many other biological tissues, exhibit a non-linear and anisotropic mechanical response to external loading. The differences in strain data between the radial and circumferential directions, as shown in Fig. 5a, do not necessarily imply that the corresponding stresses are also different. In this problem, we aim to estimate the stresses in the radial and circumferential directions for both intact and post-rupture valve leaflets.

To begin, recall that the Second Piola–Kirchhoff stress tensor ${\textbf {S}}$ is related to the strain energy density function *W* and the Lagrangian strain tensor ${\textbf {E}}$ via the following equation:

$${\textbf {S}} = \frac{\partial W({\textbf {E}})}{\partial {\textbf {E}}}.$$

Assuming that we align our coordinate system with the circumferential and radial directions of the valve, and that shear components are negligible, use the shear-free strain energy density function and the experimentally quantified material properties, both of which can be found in our previous publication [34], to derive a relationship between stress and strain in the septal leaflet.

After establishing this relationship, assume for simplicity that the values provided in Fig. 5a represent Lagrangian strains (while they are technically Eulerian strains, we will treat them as Lagrangian for the sake of this example). What are the estimated values of the Second Piola–Kirchhoff stresses in the radial and circumferential directions?

## Footnotes

1 $^{\dagger }$Greenhouse–Geisser–corrected *p*-value.

2 $^{\dagger }$Greenhouse-Geisser-corrected *p*-value.
