# Supplementary figures and images for: Chordae Rupture Alters Tricuspid Valve Leaflet Biomechanics
Source: Cardiovasc Eng Technol. 2026 Jan 5;17(2):223–36. doi: 10.1007/s13239-025-00815-9 (PMC13102865; doi:10.1007/s13239-025-00815-9)

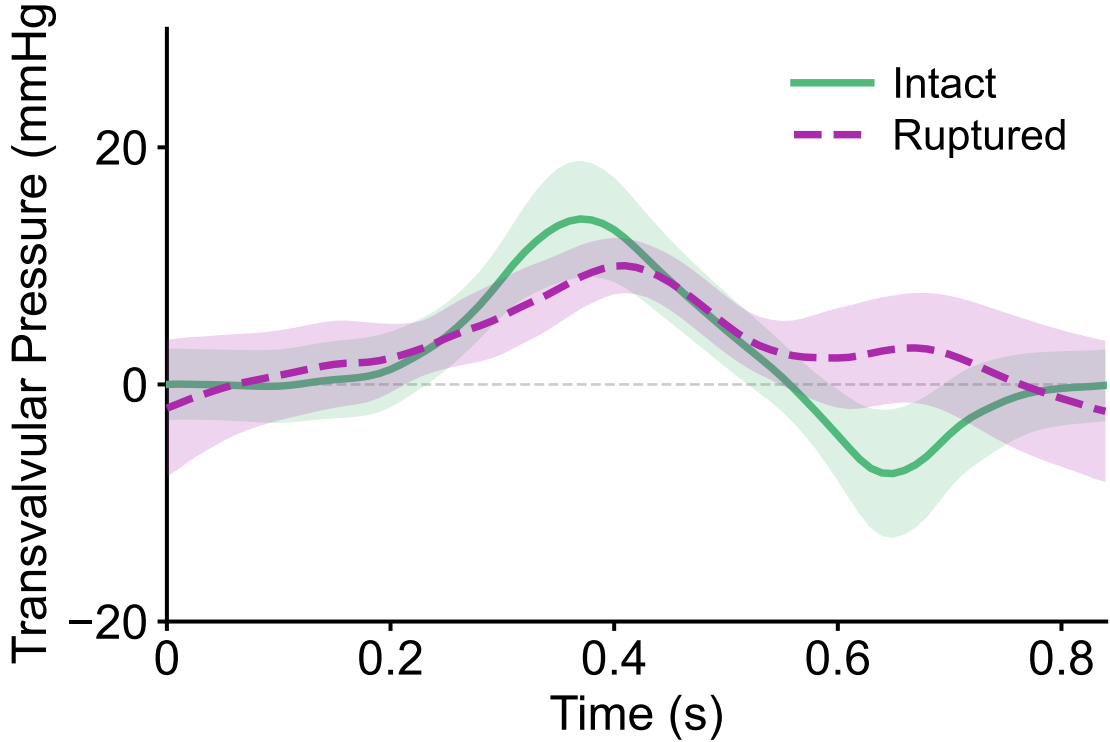

Supplement: Supplementary file 2 — (PDF 17 kb) [file 13239_2025_815_MOESM2_ESM.pdf]
